# Effects of Leaf Wetness Duration, Temperature, and Host Phenological Stage on Infection of Walnut by *Xanthomonas arboricola* pv. *juglandis*

**DOI:** 10.3390/plants12152800

**Published:** 2023-07-28

**Authors:** Concepció Moragrega, Isidre Llorente

**Affiliations:** Laboratory of Plant Pathology, Institute of Food and Agricultural Technology–CIDSAV–XaRTA, University of Girona, 17003 Girona, Spain; isidre.llorente@udg.edu

**Keywords:** disease management, epidemiology, forecasting model, walnut bacterial blight, *Xanthomonas arboricola* pv. *juglandis*

## Abstract

Bacterial blight, caused by *Xanthomonas arboricola* pv. *juglandis*, is a significant disease affecting walnut production worldwide. Outbreaks are most severe in spring, and closely tied to host phenology and weather conditions. Pathogen infections are mainly observed in catkins, female flowers, leaves, and fruits. In this study, the effect of wetness duration and temperature on walnut infections by *X. arboricola* pv. *juglandis* was determined through two independent experiments conducted under controlled environmental conditions. The combined effect of both climatic parameters on disease severity was quantified using a third-order polynomial equation. The model obtained by linear regression and backward elimination technique fitted well to the data (*R^2^* = 0.94 and *R^2^_adj_* = 0.93). The predictive capacity of the forecasting model was evaluated on pathogen-inoculated walnut plants exposed to different wetness duration–temperature combinations under Mediterranean field conditions. Observed disease severity in all events aligned with predicted infection risk. Additionally, the relationship between leaf and fruit age and the disease severity was quantified and modelled. A prediction model, which has been referred to as the WalBlight-risk model, is proposed for evaluation as an advisory system for timing bactericide sprays to manage bacterial blight in Mediterranean walnut orchards.

## 1. Introduction

Bacterial blight of Persian (English) walnut (*Juglans regia* L.), caused by *Xanthomonas arboricola* pv. *juglandis* (Pierce) Vauterin et al. [1], is an economically important disease in walnut producing areas worldwide [2,3]. In addition, *X. arboricola* pv. *juglandis* has been recently associated with two emerging diseases in walnut: vertical oozing canker [4] and brown apical necrosis [5]. Severe yield losses have been reported in Mediterranean walnut orchards due to *X. arboricola* pv. *juglandis* infections, as a consequence of premature fruit drop and shell staining of infected nuts that remain on the tree. Intense tree defoliation can also contribute to the loss of yield [6,7,8].

Infections occur in all aerial walnut organs, including catkins, female flowers, leaves, and fruit. The presence of necrotic lesions on the leaves, fruit, and twigs are characteristic disease symptoms. Leaf lesions consist of small water-soaked spots, surrounded by chlorotic halos that expand to become brown necrotic lesions. Fruit lesions are initially small water-soaked spots that progress to necrosis of the pericarp and inner nut tissues, causing premature fruit drop. Infections that occur after shell hardening usually only affect the epicarp [9].

*X. arboricola* pv. *juglandis* overwinters in walnut buds, catkins, and twig cankers [10,11], which act as a reservoir of inoculum for leaves and nutlet infections [12,13]. Populations of *X. arboricola* pv. *juglandis* present on dormant buds are the primary inoculum for nut infections [13]. Newly infected tissues can be secondary sources of inoculum. Pollen released from infected catkins plays a role in pathogen dissemination [13,14]. The bacterium is spread by moisture, especially by the combined action of wind and rain [15].

Crop losses depend on weather conditions and pathogen populations, as well as walnut cultivar. Most severe outbreaks occur in spring, highly related to host phenology and weather conditions. Epidemiological studies on walnut orchards carried out in California revealed that inoculum efficiency depends on the number of rain events following bud break. In addition, the disease pressure is closely related to population levels of *X. arboricola* pv. *juglandis* in buds and rainfall events in early spring [13]. Recently, a quantitative PCR was developed to estimate the load of pathogen cells in infected fruits as a measure of pathogen fitness to colonize the host [16].

The presence of surface wetness is crucial for the development of walnut blight, as *X. arboricola* pv. *juglandis* requires free water to infect walnut [17]. The success of pathogen infection is determined by the water congestion status of host tissues and the opening of stomata [17]. The soil characteristics and crop management might also have some influence in disease incidence and severity [18].

Current walnut blight disease management strategies involve the application of copper-based biocides alone or in combination with fungicides registered for walnut use, at regular intervals after bud break [15,17]. Conventional copper spray schedules for walnut blight control in Mediterranean orchards are based on five to seven applications from bud break until harvest. However, multiple copper sprays are not always effective in disease control [9,19], and development of resistance of *X. arboricola* pv. *juglandis* to copper has been reported due to its long-term use [20]. Additionally, regulatory restrictions on the use of copper-based compounds, especially in Europe—regulation (EU) 2018/1981—demand a better understanding of disease epidemiology that permits a reduction of copper-based applications and an increase in walnut blight management efficacy. Our previous studies demonstrated that a spray schedule based on walnut phenological stages resulted in up to four fewer copper applications—50% reduction—which led to a decrease in copper residues in the soil. Despite the decrease in the number of copper applications, the disease control was similar to or higher than that achieved through conventional strategies [9].

The efficacy of phenology-based sprays for walnut blight management could be improved if guided by a disease forecaster based on climatic parameters conducive to *X. arboricola* pv. *juglandis* infections. In this sense, a walnut blight forecast system—XanthoCast™—based on analyses of environmental data and disease progression was developed in California [21]. This model predicts *X. arboricola* pv. *juglandis* infections on walnut on the basis of wetness duration and temperature. Until now, no walnut blight forecaster developed specifically for Mediterranean orchards has been published. Moreover, most information on the influence of host phenology and climatic parameters on *X. arboricola* pv. *juglandis* infections on walnut is derived from field observations.

The present work was aimed at quantifying the effects of host phenology and climatic parameters on walnut blight disease under controlled environmental conditions in order to develop empirical models for predicting *X. arboricola* pv. *juglandis* infections on walnut. The resulting empirical model for predicting the risk of walnut blight disease based on wetness and temperature, named the WalBlight-risk model, was validated in a Mediterranean experimental orchard.

## 2. Results

### 2.1. Effect of Fruit and Leaf Age on Disease Severity

**Figure 1:**
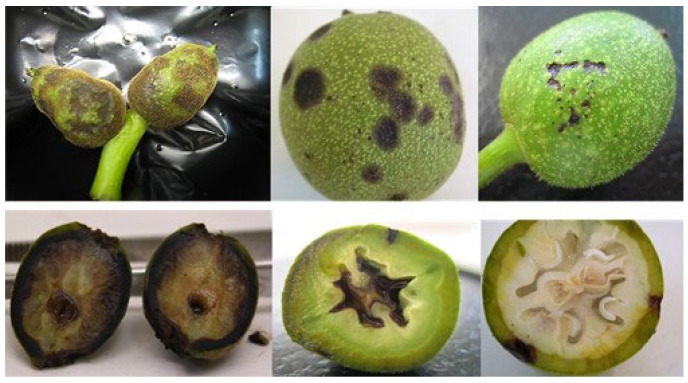
External (**top**) and internal (**bottom**) symptoms of bacterial blight on walnut fruit of different phenological stages observed fourteen days after inoculation with *X. arboricola* pv. *juglandis*. Fruit age increases from (**left**) to (**right**).

Inoculated fruits of walnut cultivars Chandler and Vina showed external water soaking and necrotic lesions. Initial infections consisted of small circular dark brown or blackish spots, which enlarged to more irregular brown lesions. Disease development through inner fruit tissues varied depending on fruit age. Lesions on immature fruits—phenological stages from Gf to Gf + 30—affected epicarp and mesocarp and progressed to the endocarp and seed (Figure 1). When fruit shell sclerification was completed—phenological stages above Gf + 45—infections were restricted to external tissues.

**Figure 2:**
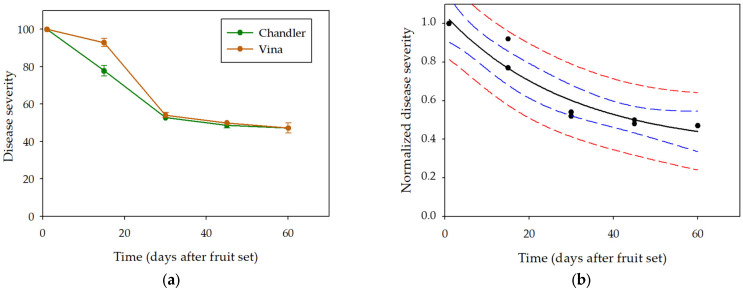
Effect of fruit age on walnut bacterial blight severity. (**a**) Mean disease severity on cvs. Chandler, and Vina walnut fruit inoculated with *X. arboricola* pv. *juglandis* at different phenological stages. Values are the mean of three replicates of three fruits per replicate and two independent experiments. Bars correspond to the mean standard error. (**b**) Three parameter–negative exponential model fitting (black line) to experimental data (dots), 95% confidence band (blue), and 95% prediction band (red) are shown.

Accordingly, susceptibility of walnut fruit to *X. arboricola* pv. *juglandis* infection decreased with fruit age in both cultivars (Figure 2a). Fruit collected at fruit set (Gf) showed maximum (100) disease severity. Similar high severities were observed on ‘Vina’ and ‘Chandler’ walnut fruit collected fifteen days after fruit set (90 and 80 respectively). Disease severity decreased to 50 when fruit age increased to GF + 30 and Gf + 60. In fact, fruit susceptibility to *X. arboricola* pv. *juglandis* remained moderate and similar for ages above GF + 30.

Analysis of covariance was performed to evaluate the effects of experiment replicate, walnut cultivar, fruit age, and their interactions in disease severity. Fruit age—days after fruit set—was defined as the covariate variable. A significant effect of fruit age (*p* < 0.0001; R^2^ = 0.82) was obtained, whereas no significant effect of experiment replicate, walnut cultivar, and factor interactions were observed. Therefore, data from two experiments and both walnut cultivars were combined to fit the general negative exponential model to the data.

Standardized disease severity (Ss = S/100) was used to fit the model. A three parameter–negative exponential model (Equation (2)) fitted well to the data (RSS = 0.0704; Adjusted R^2^ = 0.92), with the following equation: Ss = 0.35 + 0.69 *×* e^(−0.03t)^ (Figure 2b). All parameters in the model were significant (*p* < 0.001). According to this equation, the predicted minimum disease severity—in a 0–1 range—was 0.35, and the decrease rate of severity through time was 0.03.

**Figure 3:**
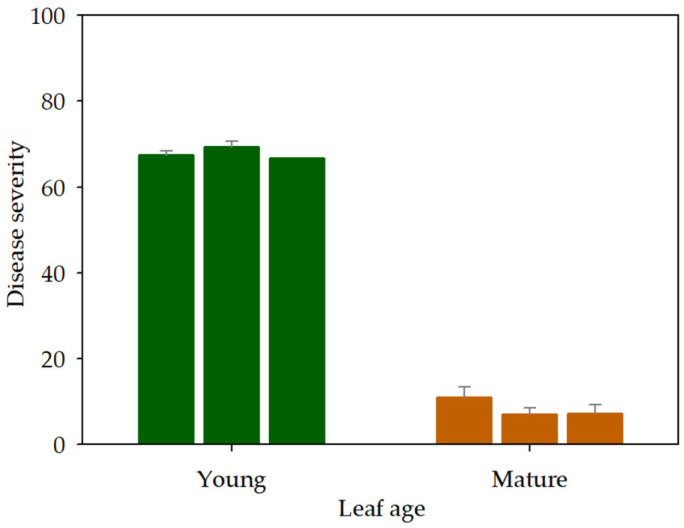
Effect of walnut leaf age on bacterial blight severity. Walnut cv. Chandler plants with young and mature leaves were inoculated with suspensions of *X. arboricola* pv. *juglandis*. Severity was assessed ten days after bacterial inoculation. Values are the mean of five replicates. Three independent experiments were performed. The mean standard error is presented.

Regarding leaf susceptibility, *X. arboricola* pv. *juglandis* inoculated ‘Chandler’ walnut leaves displayed typical bacterial blight symptoms, corresponding to small necrotic lesions sometimes surrounded by a chlorotic halo. The leaf surface affected by necrotic lesions was higher in young leaves, which became chlorotic through time. Disease severity in young walnut leaves was 65~70 in the three experiment replicates (Figure 3), whereas it decreased drastically to about 9 in mature leaves of the same walnut potted plants. No significant effect of experiment replicate was observed according to ANOVA, whereas leaf age had a high significant effect (*p* < 0.0001; R^2^ = 0.99).

### 2.2. Combined Effect of Leaf Wetness and Temperature on Disease Severity

No lesions were observed on non-inoculated plants, and few infections (7~9 disease severity) occurred in some inoculated plants incubated without leaf wetness (0 h wetness). These background levels of severity, which might be due to residual wetness after inoculation, were subtracted from disease severity data of all plants in an experiment. Disease severity ranged from 0 to 90 (Experiment 1) or to 80 (Experiment 2).

**Figure 4:**
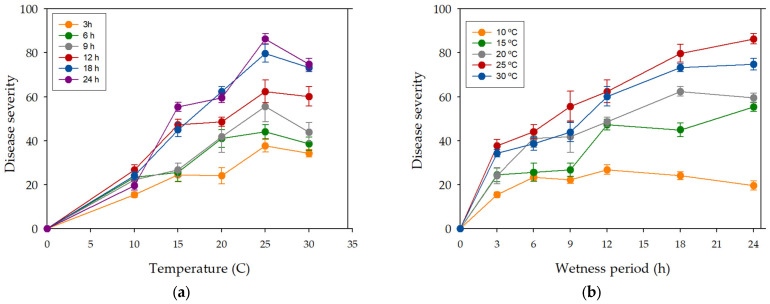
Effect temperature (**a**) and leaf wetness period (**b**) on disease severity caused by *X. arboricola* pv. *juglandis* on walnut cv. Chandler plants ten days after inoculation in two independent experiments. Values are the mean disease severity of two independent experiments and three replicates of five plants per experiment. Error bars represent the mean standard error.

The optimal range of temperature for bacterial infection was from 20 °C to 30 °C, with an experimental maximum at 25 °C (Figure 4). For temperatures from 15 °C to 30 °C, an increase in wetness period duration resulted in a gradual increase in disease severity, while, at 10 °C, low levels of disease severity (20) were observed whatever the length of wetness period. Moderate disease severity (40~50) was observed on walnut plants incubated at 15 °C under long wetness periods (18–24 h). Disease severity at 20 °C and 30 °C for long wetness periods reached 60~70. The highest disease severity was observed at 25 °C under 12–24 h continuous wetness (Figure 4).

A short wetness duration period (3 h) resulted in low disease severity, up to 20 at temperatures from 10 °C to 20 °C and from 30 to 40 at 25 °C and 30 °C. Disease severity of plants incubated above 10 °C increased with increasing wetness duration. Maximum disease severity at 15 °C was observed at 24 h wetness. Similarly, at higher temperatures (20 °C, 25 °C and 30 °C), disease severity increased progressively when increasing the wetness period duration, from 40 at 6 h wetness to 60~70 at 18–24 h wetness (20 and 30 °C) and to 86 at 24 h wetness (25 °C) (Figure 4a). Six- to nine-hour wetness duration was enough for *X. arboricola* pv. *juglandis* to produce moderate disease severity (50) in plants incubated at 25 °C, whereas 12 h of wetness were needed at 15 °C, 20 °C, and 30 °C (Figure 4b). Disease severity in walnut plants incubated at 20 °C and 30 °C under 18–24 h continuous wetness was lower than disease severity in plants incubated at 25 °C. In conclusion, wetness was required for infection of walnut by *X. arboricola* pv. *juglandis*, and disease severity was affected by temperature during the wetness period.

Relative disease severity (S’; from 0 to 1) was used to compare the two experiment replicates. No significant differences were observed between the two independent experiments according to ANOVA (*p* = 0.8291); therefore, the pooled data from two experiments were used in the model development.

Estimated parameters of a third-order polynomial model in two variables—temperature and wetness duration—are shown in Table 1. A backward stepwise selection method was used, and only parameters significantly different from zero at *p* < 0.05 were left in the model. The best accuracy in prediction when variables were removed from the pool of already selected variables was measured by F-statistic, R-Square, adjusted R-square, and Cp Mallows’ statistic (Table 1). Accordingly, the regression procedure by the backward method for model selection gave a model with a high goodness of fit to data.

**Figure 5:**
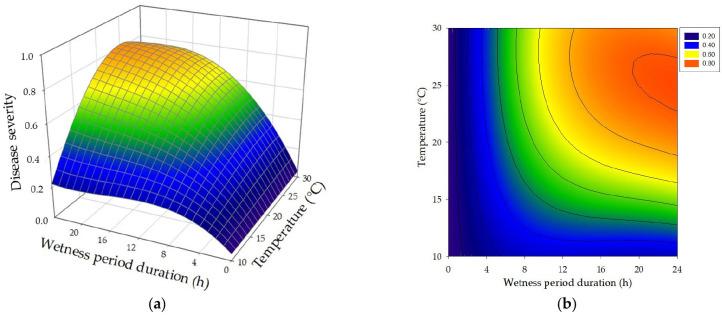
Predicted three-dimensional response (**a**) and contour plot (**b**) of the combined effects of temperature and leaf wetness duration on relative disease severity caused by *X. arboricola* pv. *juglandis* on walnut plants. Predicted values were calculated with third-order polynomial model in two variables, displayed in Table 1. Lines in contour plot (**b**) represent 0.1 increases in relative disease severity.

The surface response of the combined effects of temperature and leaf wetness duration on relative disease severity caused by *X. arboricola* pv. *juglandis* on walnut plants generated by the third-order polynomial model is shown in Figure 5a. The contour plot showed the increase in relative severity from low (<0.2) to moderate (0.2–0.5), high (0.5–0.8), and very high (>0.8) when increasing the wetness duration depending on the temperature (Figure 5b). Expected relative disease severity was maximal at 25.10 °C after 24 h of wetness.

**Figure 6:**
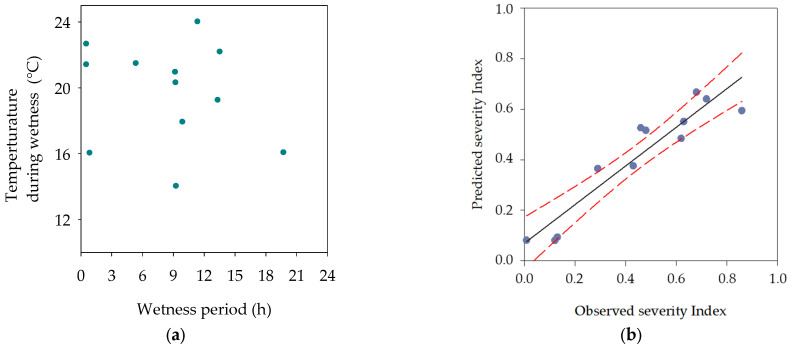
(**a**) Combined wetness period duration and mean temperature during the wetness period for model validation in twelve independent trials. (**b**) Observed disease severity index versus predicted disease severity index by the third-order polynomial model in each trial. Regression equation: *y* = 0.064 + 0.764*x* (R^2^ = 0.88; *p* < 0.001). Linear regression (black) and 95% confidence intervals (red) are presented. Walnut plants inoculated with *X. arboricola* pv. *juglandis* were exposed for 24 h under field conditions in an experimental orchard. Five plant replicates were used in each trial.

The model was evaluated for prediction of disease severity on inoculated potted walnut plants exposed to Mediterranean field conditions from May to July in Girona (Spain). The wetness duration period ranged from 0.5 h to 20 h and the mean temperature during the wetness period ranged from 14 °C to 24 °C (Figure 6a). A total of twelve independent trials were performed, in which low, moderate, and high levels of predicted disease severity were included.

The observed disease severity index ranged from 0 to 0.8, depending on the validation trial. The duration of wetness in each 24 h period and the mean temperature observed during the wetness period were interpolated on the equation previously described (Table 1), and a predicted severity index was calculated. The observed severity index was compared with model predictions for the same wetness–temperature conditions (Figure 6b).

The linear regression of the predicted severity indexes against the observed severity indexes showed a high relationship between both variables (Figure 6b), which confirmed the good accuracy of the developed model. The coefficients of determination R^2^ and adjusted R^2^ for the linear regressions were both 0.88 (*p* < 0.001). The intercept was not significantly different from zero (*p* = 0.167). Neither significant underprediction nor overprediction of the model was observed for the whole set of data (Figure 6b).

On the basis of the disease severity index (I), model predictions agreed on all 12 cases (100%); 3 for low (I < 0.2), 2 for moderate (0.2 ≤ I < 0.5), and 7 for high (I ≥ 0.5) severity predictions.

## 3. Discussion

Occurrence and development of plant diseases are influenced by the interaction of the host plant, the pathogen, and the environmental conditions. Phenological stage of development and cultivar susceptibility have been identified as key host determinants in walnut bacterial blight disease development [12].

Several field studies have been carried out to assess walnut cultivar susceptibility to bacterial blight in Mediterranean orchards [22,23]. Differential susceptibility to bacterial blight depending on walnut cultivar has been observed, which does not always coincide in different geographical areas [22,23,24]. In a five-year field evaluation for blight susceptibility in northeastern Spain, walnut cultivars Franquette and Chandler showed low susceptibility to blight [23]. Interestingly, Franquette is considered moderately susceptible to blight in French walnut orchards [25]. On the other hand, these cultivars showed a high level of susceptibility in artificial inoculation tests on immature fruit [23]. Therefore, cultivars may appear to differ in their susceptibility to infection by *X. arboricola* pv. *juglandis* under field conditions. This variation might be explained by the availability of natural inoculum and its heterogeneous populations, the changing environmental conditions, and the phenological stage of the host plant [26].

To prevent variation of walnut response to bacterial blight, experiments with artificial inoculation of *X. arboricola* pv. *juglandis* are proposed under field [27,28] or controlled environment conditions [7,23]. In the present work, ex vivo and in planta assays performed under controlled conditions proved useful for determining the effects of host phenology and climatic parameters on walnut infection by *X. arboricola* pv. *juglandis*, since they only reflect host susceptibility at the genetic level.

Our results confirmed that cultivars Chandler and Vina are highly susceptible to *X. arboricola* pv. *juglandis*, in agreement with previous studies [17,23], thus demonstrating the potential of these cultivars to be infected by *X. arboricola* pv. *juglandis* under conditions favorable for the disease. The use of two highly susceptible cultivars made it possible to compare the host response to bacterial blight infections at different leaf and fruit growth stages.

A significant effect of walnut fruit and leaf age on bacterial blight severity was observed. Young leaves and immature fruit were highly susceptible. Disease severity on the youngest fruit—Gf to Gf + 15—was high (80~100). Moreover, immature fruit aged up to 30 days after fruit set (Gf) developed lesions that progressed from external tissues to the seed. These results support field observations on walnut orchards, where severe fruit lateral or apical infections are reported in spring during the first half of nut growth [17,18]. Field infections that occur prior to shell hardening rot or shrivel the developing kernel, making fruit unmarketable or causing premature fruit drop [9]. These fruit infections that occurring soon after flowering produce most of the economic losses. Interestingly, our results demonstrated that, despite a decrease in fruit susceptibility, infections of moderate severity were observed on fruit aged above Gf + 45, which developed lesions confined to the husk. In agreement, fruit infections have been reported under favorable field conditions later in the growing season. These affected fruit remain on the tree until mature, and a brown stain on the shell is often observed after husk removal [3].

The variation in walnut bacterial blight disease severity as a function of fruit age was modelled. A three parameter–negative exponential model was developed, which fitted well to the experimental data. The model predicts potential fruit severity (in a 0–1 range) based on fruit age (days after fruit set). The predicted minimum disease severity by the model equation is 0.35, explained by the moderate susceptibility of mature fruit to *X. arboricola* pv. *juglandis* infections.

Regarding to leaf susceptibility, mature walnut leaves were demonstrated to be significantly less susceptible to *X. arboricola* pv. *juglandis* than the young ones, with a reduction of 90% in disease severity. Leaf infections in walnut orchards generally occur from mid to late spring, and provide the inoculum for fruit infections [3]. These results give evidence of differential host susceptibility among plant organs within the same cultivar. Contents of phenolics implicated in defense against walnut blight may partially explain this differential susceptibility of fruit and leaves of susceptible walnut cultivars depending on the organ and the phenological stage [25,29,30,31].

The development of walnut bacterial blight disease is highly dependent on environmental conditions, especially in spring. Great differences in disease incidence were observed in dry spring years (10% incidence) compared to wet spring years (>90% incidence) [17]. Rainfall, wetness, and temperature conditions critically determine the disease occurrence and progression [19,32]. In this work, experiments performed under controlled environmental conditions permitted us to determine the effects of wetness period duration and mean temperature during the wetness period on disease severity under host and pathogen favorable conditions—susceptible cultivar, young leaves, and high concentration of inoculum.

The combined effect of leaf wetness duration and temperature on infection of walnut by *X. arboricola* pv. *juglandis* was analyzed for temperatures from 10 °C to 30 °C, which encompasses the range of temperature observed in Mediterranean orchards during the walnut growing season. Results confirmed that wetness is required for bacterial infection on walnut plants. Optimal temperatures for walnut infection by *X. arboricola* pv. *juglandis* conducive to severe disease (S’ > 0.5) ranged from 20 °C to 30 °C, with a maximum at 25 °C. At lower temperatures (10–15 °C) the pathogen was able to infect, although low (20 at 10 °C) or moderate (up to 40~50 at 15 °C) disease severity was observed.

In Mediterranean walnut orchards, average temperatures of 10–15 °C are often observed at the beginning of the growing season. In addition, rainfall events or wet periods are more frequent in spring than in summer. According to results derived from this work, three hours of wetness is enough for bacterial infection at temperatures from 10 to 15 °C, although low disease severity was observed. It is important to note, however, that wetting periods of 12–24 h at 15 °C resulted in moderate disease severity. These findings are consistent with field studies of walnut bacterial blight disease epidemiology. *X. arboricola* pv. *juglandis* infections in walnut orchards occur soon after flowering [8]. Studies performed in Californian walnut orchards revealed that 12–24 h of wetness was required at temperatures from 15 to 25 °C for disease development on up to ten-week-old fruit [33]. Moreover, it has been reported that final walnut blight incidence is correlated positively to the quantity of rainfall during the first 4 weeks after bud-burst [18]. The highest susceptibility of immature fruit and leaves may explain why these spring infections result in high economic losses. Similarly, early-leafing varieties are most severely affected by the disease, explained by the exposure to more frequent periods of surface wetness than those that develop later in the season [17].

According to the present study, at the optimal temperatures for infection (20–30 °C), three-hour wetness periods are conducive to moderate disease severity and, when wetness duration increases from nine to twenty-four hours, the disease severity is high. Thus, if wet or rainy periods occur at optimal temperatures for infection in the late spring or early summer, the epidemics may be severe. In fact, results from experiments on the effects of host age on disease severity indicated that, under climatic conditions conducible to bacterial infections, mature fruit might be infected by the pathogen, although lesions remain restricted to external fruit tissues, whereas infections in mature leaves should be less frequent. However, in Mediterranean orchards, new infections are rare during summer because the conditions for them to occur are less frequent [8].

The model obtained in this work, hereinafter referred to as the WalBlight-risk model, accurately described the relationship between wetness duration and temperature on disease severity, according to predicted and observed comparisons for disease severity index. Predictions were consistent with field observations.

Several attempts to develop walnut blight prediction models have been performed based on field studies. XanthoCast™ was developed as a model to predict infection periods for walnut blight by the University of California based on analyses of environmental data and disease progression over several seasons and locations [33]. The XanthoCast™ model was evaluated and refined, and it is used as a support system by growers in spray timing for walnut blight disease management in California (USA) [34]. Similarly, walnut bacterial blight progression in commercial orchards through three years was modelled based on disease incidence on half full-size diameter fruit of Vina walnuts in Tasmania [17]. Timing bactericide sprays for walnut blight management based on these forecasting models seem to be more efficient in dry years than in wetter years [17,35]. On the basis of the available literature, these models have not been evaluated in walnut orchards in the Mediterranean region. In the present work, the WalBlight-risk model was empirically developed under high inoculum pressure and optimal host susceptibility. To our knowledge, this is the first walnut blight forecasting model developed under controlled environmental conditions in which the combined effects of a wide range of wetness duration periods (0 to 24 h) and temperatures (10 to 30) were evaluated. Moreover, the WalBlight-risk model was validated under Mediterranean field conditions. It would be interesting to evaluate its accuracy to predict the infection events and the disease severity under field conditions in dry and wet years.

Finally, a reduced spraying program for walnut blight disease control in Mediterranean orchards resulted in similar or even better efficacy than the standard program [9]. This reduced spraying schedule consisted of three copper-based compound applications in early spring, which omitted the last four summer treatments in the standard schedule. The efficacy of this early season schedule could be improved if guided by a forecasting model based on climatic parameters. The WalBlight-risk model developed to predict walnut blight severity based on combined effects of wetness duration and temperature could be used to support bactericide sprays for disease management in Mediterranean walnut orchards, once evaluated in field trials. The WalBlight-risk model can also be used for additional purposes, such as disease risk assessment, to design disease management strategies, and scenario analysis—predicting the effects of climate change on the epidemiology of walnut blight.

## 4. Materials and Methods

### 4.1. Plant Material

Self-rooted cv. Chandler walnut potted plants obtained by micropropagation (Vitrotech Vitrotech^®^ S.A, Murcia, Spain) were used. Plants were grown in a greenhouse and fertilized once a week with a solution of 200 ppm N–P–K (20–10–20). Walnut fruit of cvs. Chandler and Vina were collected from the walnut collection of IRTA–Mas Badia (Canet de la Tallada, Girona, Spain).

### 4.2. Pathogen

*Xanthomonas arboricola* pv. *juglandis* strain 1330.3c isolated from lesions in walnut fruit from Zaragoza (Spain) [36], provided by Instituto Valenciano de Investigaciones Agrarias (Moncada–Valencia, Spain), was used. Bacterial strain was stored at −70 °C in 20% wt/vol glycerol yeast–peptone–glucose broth stock tubes, and routinely grown at 27 °C on Yeast–Peptone–Glucose Agar (YPGA) [37]. Bacterial suspensions in sterile distilled water were prepared from 48-hour-old cultures grown at 27 °C on Luria–Bertrani (LB) plates. Inoculum concentration was adjusted to an optical density of 0.2 at 600 nm (1–5 × 10^8^ CFU/mL).

### 4.3. Effect of Fruit and Leaf Age on Susceptibility to Disease

Walnut fruit of cultivars Chandler and Vina, collected at phenological stages GF (fruit set), GF + 15, GF + 30, GF + 45 and GF + 60, were surface disinfected by immersion in 5% sodium hypochlorite solution for 1–2 min and rinsed three times with sterile distilled water. Then, fruit were left to surface dry, and dipped for 1 min into the bacterial suspension (1–5 × 10^8^ CFU/mL). Inoculated fruit were placed on wet filter paper in plastic boxes, sealed in moistened transparent polyethylene bags—to maintain high RH—and incubated at 25 °C and 16 h light photoperiod for 10–15 days in a controlled environment chamber (model MLR–350; Sanyo, Gunma, Japan). At the end of the incubation period, the disease severity was assessed according to a 0–4 severity index (I) corresponding to 0: no symptoms, and up to 10%, 30%, 60%, and 100% fruit surface covered by necrotic spots, respectively.

Self-rooted cv. Chandler potted walnut plants were pruned and forced to branch one month before the experiments, so that mature and young leaves were present in the same twig. Bacterial suspension (1~5 × 10^8^ CFU/mL) supplemented with diatomaceous earth (1 mg/mL) of strain *X. arboricola* pv. *juglandis* 1330.3c was sprayed to run off on all leaves in a plant with a compressed air atomizer operated at 2 kP. Inoculated walnut plants were placed in moist chambers—moistened plastic bags—under darkness for 24 h, then plastic bags were removed and plants were transferred to a plant growth chamber (PGR15 Conviron, Winnipeg, MB, Canada) and incubated for ten days at 25 °C, 70–80% RH, and 16 h light photoperiod for disease development. A 0-to-4 scale severity index was used, corresponding to a leaf area affected by zero, 1–5, 6–25, 26–50, and 51–100% necrosis, respectively.

Disease severity (S) was calculated for each individual plant or fruit replicate according to the Equation (1).

(1) $$S=\left[(\sum_{i=1}^{N}I_{n})/(N\times 4)\right]\times 100$$

where I*_n_* is the severity index for each leaf or fruit, *N* is the number of leaves with the same age per plant or fruit in a replicate, and four is the maximum severity index value.

Plants and fruit inoculated with sterile distilled water were included as negative controls. A complete randomized experimental design was used with three replicates of three fruits or five plants per replicate. The fruit experiments were performed twice, and plant assays were conducted three times.

The effect of factors—cultivar, age and trial in fruit assays, and age and trial in plant assays—was determined using the GLM procedure of SAS software version 9.4 (SAS Institute Inc., Cary, NC, USA) after confirmation of the homogeneity of variance and normality.

A general negative exponential model was fitted to disease severity–fruit age data by nonlinear regression. The NLIN procedure of SAS software version 9.4 was used (SAS Institute Inc., Cary, NC, USA). The model (Equation (2)) describes fruit disease severity (Sf) as an exponential function of fruit age (*x*); *y*_0_ represents the asymptote to which the disease severity converges; *a* is the difference between Y maximum and asymptote; and *b* indicates the severity decrease rate.

(2) $$Sf=y_{0}+ae^{-bx}$$

The goodness of fit of the model was assessed using the residual sum of squares (RSS).

### 4.4. Modelling the Combined Effect of Wetness Period Duration and Temperature on Disease Severity

Bacterial suspensions (1–5 × 10^8^ CFU/mL) supplemented with diatomaceous earth (1 mg/mL) were sprayed on one- to two-year-old, thirty-centimeter-high cv. Chandler walnut potted plants with young expanded leaves. Adaxial and abaxial leaf surfaces were inoculated to run off with a compressed air atomizer operated at 2 kP. Plants inoculated with distilled water amended with diatomaceous earth (1 mg/mL) were used as negative controls. Inoculated plants were introduced into wet plastic bags to maintain leaf wetness, and incubated at 10, 15, 20, 25, or 30 °C (±1 °C) under darkness in controlled environment chambers (model MLR–350; Sanyo, Gunma, Japan). At 0, 3, 6, 9, 12, 18, or 24 h time intervals, plastic bags were removed and plants were transferred to a plant growth chamber (PGR15 Conviron, Winnipeg, MB, Canada) and incubated at 25 °C, 70–80% RH, and 16 h light photoperiod for disease development. Environmental variables were continuously monitored within the chambers with a datalogger (CR10, Campbell Scientific Ltd., Loughborough, UK) connected to combined temperature–relative humidity (model HMP35C) and leaf wetness (model 237) sensors. All temperature–wetness period combinations were tested.

Disease severity was assessed in all young leaves in a plant ten days after inoculation. A 0-to-4 scale severity index (I) was used, corresponding to a leaf area affected by 0, 1–5, 6–25, 26–50, and 51–100%, respectively. Disease severity (S) was calculated for each plant according to the formula described in Equation (1). A complete randomized design was used. Each treatment—temperature–wetness period—consisted of three replicates of five plants per replicate. The experiment was conducted twice. Disease severity of each plant was standardized using the maximum value observed in each experiment. The relative disease severity (S’ = S/Smax) ranged from zero to one, so both experiment replicates were compared. The effect of experiment replicate was determined by analysis of variance, after confirmation of the homogeneity of variance and normality, using the general linear models (GLM) procedure of SAS software version 9.4 (SAS Institute Inc., Cary, NC, USA).

As there were no significant differences between the two independent experiments for relative disease severity, the effect of temperature (T) and wetness duration (W) on relative disease severity (S’) was estimated with averaged data of two experiments by regression analysis. The following model was tested: S’ = f (T, W), in which S’ is the relative disease severity per replicate, and f (T, W) is a linear function of the terms W, T, WT, T^2^, W^2^, T^2^ W, TW^2^, T^2^ W^2^, T^3^, T^3^ W, TW^3^. The REG procedure of SAS software version 9.4 was used to fit the model to data using the backward method. A model was selected when all variables were significant at the 0.05 level. The criteria to evaluate this model were randomness and normality of residuals and model goodness of fit tests, including ANOVA (F-statistic), R^2^, adjusted R^2^, and Cp Mallows’ statistic. Mallows’ statistic Cp was used to select the best model according to the Mallows’ criterion, given that, if the model has a high accuracy, the parameter estimates are unbiased.

### 4.5. Model Validation

The capacity of the model for predicting the infection risk was determined in additional experiments performed under field conditions in a Mediterranean experimental orchard, according to Montesinos et al. [38] with modifications. Walnut potted plants cv. Chandler were artificially inoculated in the orchard with bacterial suspensions (1–5 × 10^8^ CFU/mL) as described above, and exposed to field conditions for a 24-h period—from 8:00 p.m. to 8:00 p.m. of the following day. Twelve validation trials were performed from May to July. In each trial, five walnut plants were inoculated with the pathogen and exposed to field conditions. Additionally, on each date, a set of five plants was inoculated in the laboratory with the same bacterial suspension and incubated for 24 h at 25 °C under continuous wetness as a positive control. Plants inoculated with sterile distilled water and exposed to field conditions were included as negative controls. After 24 h incubation under field/laboratory conditions, plants were left to leaf surface dry (<60 min) and incubated at 25 °C, 70–80% RH, and 16 h light photoperiod for disease development. Wetness, rainfall, and temperature were monitored with electronic sensors connected to a CR–10X datalogger (Campbell Scientific, Inc., Loughborough, UK). Disease severity was recorded ten days after incubation, as described above. In order to minimize the inoculum and plant variation among events, an index of observed disease was calculated using the ratio between the disease severity obtained under field conditions and the disease severity in laboratory-inoculated plants incubated at optimal conditions for infection, in the same validation trial. A predicted disease index was calculated for each temperature–wetness combination with the model equation. Model performance was evaluated by regression of the observation on the predicted disease index.

## 5. Conclusions

The effects of host phenology and climatic parameters on walnut blight disease were evaluated under controlled environmental conditions. Bacterial blight disease severity in walnut fruit decreases with fruit age according to a negative exponential model. Wetness is required for infection of walnut by *X. arboricola* pv. *juglandis*, with disease severity being affected by the temperature during the wetness period.

A temperature and wetness duration-based walnut blight-forecasting model, named the WalBlight-risk model, has been developed and successfully validated under Mediterranean field conditions.

## Figures and Tables

**Table 1:** Parameters of a third-order polynomial model in two variables describing the walnut bacterial blight disease severity on combined effects of temperature (T) and duration of leaf wetness (W). Model goodness of fit to experimental data is displayed.

Variable	Parameter Estimate	Standard Error	Type II SS	F Value	Pr > F
Intercept	0.04303	0.01618	0.03291	7.07	0.0101
TW	0.00441	0.00033020	0.82992	178.24	<0.0001
W^2^	−0.00285	0.00054108	0.12885	27.67	<0.0001
W^3^	0.00007549	0.00002206	0.05450	11.71	0.0011
T^3^W	−0.00000181	2.406374E−7	0.26470	56.85	<0.0001
TW^3^	−0.00000170	4.616193E−7	0.06329	13.59	0.0005
**Model goodness of fit**					
R-Square	Adj R-Square	Cp	MSE	Pr > F	
0.9349	0.9293	6.6144	0.0047	<0.0001	

## Data Availability

The data presented in this study are available on request from the corresponding author.

## References

[1] Vauterin L., Hoste B., Kersters K., Swings J. (1995). Reclassification of *Xanthomonas*. Int. J. Syst. Bacteriol..

[2] EPPO Global Database *Xanthomonas arboricola* pv. *juglandis*. https://gd.eppo.int/taxon/XANTJU/distribution.

[3] Lamichhane J.R. (2014). *Xanthomonas arboricola* Diseases of Stone Fruit, Almond, and Walnut Trees: Progress toward Understanding and Management. Plant Dis..

[4] Hajri A., Meyer D., Delort F., Guillaumès J., Brin C., Manceau C. (2010). Identification of a Genetic Lineage within *Xanthomonas arboricola* pv. *juglandis* as the Causal Agent of Vertical Oozing Canker of Persian (English) Walnut in France. Plant Pathol..

[5] Moragrega C., Matias J., Aletà N., Montesinos E., Rovira M. (2011). Apical Necrosis and Premature Drop of Persian (English) Walnut Fruit Caused by *Xanthomonas arboricola* pv. *juglandis*. Plant Dis..

[6] Loreti S., Gallelli A., Belisario A., Wajnberg E., Corazza L. (2001). Investigation of Genomic Variability of *Xanthomonas arboricola* pv. *juglandis* by AFLP Analysis. Eur. J. Plant Pathol..

[7] Vagelas L.K., Rumbos C.I., Tsiantos J. (2012). Variation in Disease Development among Persian Walnut Cultivars, Selections and Crosses When Inoculated with *Xanthomonas arboricola* pv. *juglandis* in Greece. J. Plant Pathol..

[8] Scortichini M. (2010). Epidemiology and Predisposing Factors of Some Major Bacterial Diseases of Stone and Nut Fruit Trees Species. J. Plant Pathol..

[9] Ninot A., Aletà N., Moragrega C., Montesinos E. (2002). Evaluation of a Reduced Copper Spraying Program to Control Bacterial Blight of Walnut. Plant Dis..

[10] Miller P.W., Bollen W.B. (1946). Walnut Bacteriosis and Its Control.

[11] Mulrean E.N., Schroth M.N. (1981). Ecology of *Xanthomonas campestris* pv. *juglandis* on Persian (English) Walnuts. Phytopathology.

[12] Olson W.H., Buchner R.P., Adaskaveg J.E., Lindow S.E. (1997). Walnut Blight Control in California. Acta Hortic..

[13] Lindow S., Olson W., Buchner R. (2014). Colonization of Dormant Walnut Buds by *Xanthomonas arboricola* pv. *juglandis* Is Predictive of Subsequent Disease. Phytopathology.

[14] Ark P.A. (1944). Pollen as a Source of Walnut Bacterial Blight Infection. Phytopathology.

[15] Buchner R.P., Gilles C., Olson W.H., Adaskaveg J.E., Lindow S.E., Koutsoukis R. (2010). Spray Timing and Materials for Walnut Blight (*Xanthomonas campestris* pv. *juglandis*, *Xanthomonas arboricola* pv. *juglandis*) Control in Northern California USA. Acta Hortic..

[16] Martins L., Fernandes C., Albuquerque P., Tavares F. (2019). Assessment of *Xanthomonas arboricola* pv. *juglandis* Bacterial Load in Infected Walnut Fruits by Quantitative PCR. Plant Dis..

[17] Lang M.D., Evans K.J. (2010). Epidemiology and Status of Walnut Blight in Australia. J. Plant Pathol..

[18] Chevallier A., Bray O., Prunet J.P., Giraud M. (2010). Factors Influencing Walnut Blight Symptoms Emergence and Development. Acta Hortic..

[19] Lang M.D., Hills J.L., Evans K.J. (2005). Preliminary Studies toward Managing Walnut Blight in Tasmania, Australia. Acta Hortic..

[20] Gardan L., Brault T., Germain E. (1993). Copper Resistance of *Xanthomonas campestris* pv. *juglandis* in French Walnut Orchards and Its Association with Conjugative Plasmids. Acta Hortic..

[21] Adaskaveg J.E., Förster H., Thompson D., Enns J., Connell J., Buchner R. (2009). Epidemiology and Management of Walnut Blight.

[22] Tamponi G., Donati G.P. (1990). Walnut Cultivars Susceptibility to *Xanthomonas juglandis*. Acta Hortic..

[23] Aletà N., Ninot A., Moragrega C., Llorente I., Montesinos E. (2001). Blight Sensitivity of Spanish Selections of *J. regia*. Acta Hortic..

[24] Arquero O., Lovera M., Rodriguez R., Salguero A., Trapero A. (2005). Characterization and Development of Necrotic Lesions of Walnut Tree Fruits in Southern Spain. Acta Hortic..

[25] Radix P., Bastien C., Jay-Allemand C., Charlot G., Seigle-Murandi F. (1998). The Influence of Soil Nature on Polyphenols in Walnut Tissues. A Possible Explanation of Differences in the Expression of Walnut Blight. Agronomie.

[26] Solar A., Colaric M., Hudina M., Štampar F. (2005). Phenolic Content of Walnut Fruit as Affected by Cultivar and Developmental Stage. Acta Hortic..

[27] Adaskaveg J.E., Förster H., Thompson D., Cary D., Wade L., Dandekar A., Brown P.J., Leslie C. (2021). Epidemiology and Management of Walnut Blight. Walnut Res. Rep..

[28] Woeste K.E., McGranahan G.H., Schroth M.N. (1992). Variation among Persian Walnuts in Response to Inoculation with *Xanthomonas campestris* pv. *juglandis*. J. Am. Soc. Hortic. Sci..

[29] Solar A., Colarič M., Usenik V., Stampar F. (2006). Seasonal Variations of Selected Flavonoids, Phenolic Acids and Quinones in Annual Shoots of Common Walnut (*Juglans regia* L.). Plant Sci..

[30] Solar A., Jakopic J., Veberic R., Stampar F. (2012). Correlations between *Xanthomonas arboricola* pv. *juglandis* Severity and Endogenous Juglone and Phenolic Acids in Walnut. J. Plant Pathol..

[31] Bujdosó G., Lengyel-Kónya É., Berki M., Végh A., Fodor A., Adányi N. (2022). Effects of Phenolic Compounds on Walnut Bacterial Blight in the Green Husk of Hungarian-Bred Cultivars. Plants.

[32] Adaskaveg J.E., Förster H., Thompson D., Cary D., Nguyen K., Connell J., Buchner R. (2013). Epidemiology and Management of Walnut Blight. Walnut Res. Rep..

[33] Adaskaveg J.E., Kirkpatrick B.C., Buchner R., Olson W.H. (1994). Epidemiology and Management of Walnut Blight. Walnut Res. Rep. 94 WMB.

[34] Adaskaveg J.E., Förster H., Dieguez-Uribeondo J., Thompson D., Thomas C., Buchner R., Olson B. (2001). Epidemiology and Management of Walnut Blight. Walnut Res. Rep..

[35] Adaskaveg J., Förster H., Thompson D., Driver G., Connell J., Buchner R. (2005). Epidemiology and Management of Walnut Blight. Walnut Res. Rep..

[36] López M.M., Martí R., Morente C., Orellana N., Ninot T., Aleta N. (1994). Bacterias Fitopatógenas Identificadas en Nogal en España. Investig. Agrar. Prod. Protec. Veg..

[37] Boudon S., Manceau C., Nottéghem J.L. (2005). Structure and Origin of *Xanthomonas arboricola* pv. *pruni* Populations Causing Bacterial Spot of Stone Fruit Trees in Western Europe. Phytopathology.

[38] Montesinos E., Moragrega C., Llorente I., Vilardell P., Bonaterra A., Ponti I., Bugiani R., Cavanni P., Brunelli A. (1995). Development and Evaluation of an Infection Model for *Stemphylium vesicarium* on Pear Based on Temperature and Wetness Duration. Phytopathology.

